# Gastric Adenocarcinoma of the Remnant Stomach After Roux-en-Y Diagnosed by Endoscopic Ultrasound-Directed Transgastric Endoscopic Retrograde Cholangiopancreatography (EDGE)

**DOI:** 10.7759/cureus.37697

**Published:** 2023-04-17

**Authors:** Ariana R Tagliaferri, Dhruv Patel, Yana Cavanagh

**Affiliations:** 1 Internal Medicine, St. Joseph's Regional Medical Center, Paterson, USA; 2 Gastroenterology, St. Joseph's Regional Medical Center, Paterson, USA

**Keywords:** eus, ercp, remnant stomach, gastric adenocarcinoma, edge, roux-en-y, gastric bypass

## Abstract

It can be difficult to evaluate for pathology with traditional endoscopic modalities following a Roux-en-Y gastric bypass. This is due to the truncated gastrointestinal tract and excluded distal stomach formed during a Roux-en-Y procedure. In these circumstances, a modified endoscopic procedure, known as endoscopic ultrasound (EUS)-directed transgastric endoscopic retrograde cholangiopancreatography (ERCP) (EDGE) is used. Although the Roux-en-Y procedure slightly increases the risk of gastric adenocarcinoma in the general population, the occurrence of gastric adenocarcinoma in the excluded stomach, specifically, is uncommon. Herein, we present a case of gastric adenocarcinoma of the excluded stomach, diagnosed 20 years after a Roux-en-Y procedure. This case is unique because after an extensive five-year workup for melena and iron deficiency anemia, the malignancy was ultimately diagnosed utilizing the innovative EDGE procedure.

## Introduction

This manuscript was previously presented as a poster at the American College of Gastroenterology Annual Conference, in October of 2022, and published as an abstract in the American Journal of Gastroenterology with the following citation: S3549. Gastric Adenocarcinoma of the Remnant Stomach After Roux-en-Y Diagnosed by EDGE. The American Journal of Gastroenterology. 117(10S):e2228, October 2022. doi: 10.14309/01.ajg.0000870836.84374.b7. It is cited in this manuscript where necessary.

Gastric adenocarcinoma is the fourth most common incidental cancer and the fifth most common malignancy worldwide [1,2]. Several risk factors are implicated in the diagnosis of gastric adenocarcinoma, such as *Helicobacter pylori* infections causing chronic inflammation of the gastric mucosa, obesity, smoking, consumption of foods rich in nitric oxide, and previous gastric surgeries [1]. Roux-en-Y surgery is a type of gastric bypass procedure in which a small pouch is created to reduce the size of the stomach by connecting the distal stomach to the small intestine [3]. This procedure minimizes caloric intake, subsequently inducing weight loss [3]. Given the limited surveillance of the excluded stomach, malignancy in this specific area tends to be diagnosed at advanced stages and often present with nonspecific symptoms [4]. The true incidence is unknown because it is so rare; however, a study from Brazil noted the incidence to be 0.03% [4]. Due to multifactorial reasons, such as increased inflammation and/or chronic pancreaticobiliary reflux, patients have a higher incidence of gastric cancer after Roux-en-Y compared to the general population [5]. However, it is important to note that the occurrence of gastric adenocarcinoma of the excluded stomach is rare [5]. We report a case where the patient presented with melanotic stools and iron deficiency anemia and was diagnosed with gastric cancer of the excluded stomach using an advanced endoscopy technique referred to as the “EDGE” procedure or endoscopic ultrasound (EUS)-directed transgastric endoscopic retrograde cholangiopancreatography (ERCP).

The EDGE procedure was developed as an easier, less invasive way to access the ampulla in patients who have previously undergone Roux-en-Y surgeries [6]. Hepatobiliary conditions are common following Roux-en-Y surgeries, and thus, an approach that would be efficacious and safe was warranted [6]. Enteroscopies were also useful for the diagnosis and management of small bowel diseases [6]. Numerous endoscopic methods have been explored over the years, including colonoscope- and enteroscope-assisted ERCP, double- and single-balloon enteroscopies, and spiral enteroscopies [6]. However, there were limitations in mobilizing the scopes due to the long and wide dimensions and the size of the channels to allow the use of stents and accessories [6]. In fact, balloon enteroscopy was only most successful in patients with short Roux limb with bilioenteric anastomosis and intact papilla [6]. The length of the Roux limbs, the presence of adhesions, and internal hernias restricted the success rates of any approach [6]. With the development of the EDGE procedure in 2013, endoscopists can now traverse and manipulate any adhesions and internal hernias that may be obstructing the views and/or impairing the ability to maneuver the scopes [6]. Additionally, by bypassing the Roux limbs and creating a gastrogastrostomy, endoscopists can not only diagnose and treat hepatobiliary diseases and small bowel diseases but also evaluate the remnant stomach [6]. Numerous clinical trials and studies have demonstrated that this approach is clinically superior due to its high technical and clinical success rates, shorter procedure times, reduced hospital costs and length of stay, and minimal chances of complications, such as bleeding, malposition, migration, perforation, and pancreatitis [6].

## Case presentation

A 67-year-old female with past medical history of hypothyroidism, generalized anxiety, major depressive disorder, cholecystectomy, obesity status post gastric bypass (Roux-en-Y approximately 20 years prior), and chronic immune thrombocytopenic purpura (ITP) status post splenectomy was transferred from an outside hospital for endoscopic ultrasound (EUS) and endoscopic retrograde cholangiopancreatography (ERCP) after admission for syncope and melena. Of note, the patient has a history of melanotic stools with iron deficiency anemia for which she had multiple admissions over the course of five years, where esophagogastroduodenoscopies and colonoscopies were performed without a clear source of bleeding. Two video capsule endoscopies were also performed, which revealed frank blood around the jejunum; however, no source of bleeding was identified. On this admission, the patient was found to have a hemoglobin of 7.5 g/dL, requiring the transfusion of two units of packed red blood cells.

She was hemodynamically stable on arrival and had a hemoglobin of 7.7 mg/dL. She underwent a computerized tomography (CT) angiogram, which showed a 5 cm sentinel clot in the proximal excluded gastric remnant stomach without evidence of hemorrhage. At this stage, the working diagnosis was acute blood loss anemia given the melanotic stools; however, prior workup with imaging and endoscopic intervention had not revealed the source of bleeding. Additionally, her iron deficiency anemia was thought to be chronic given her history of ITP. Given the lack of constitutional symptoms, such as night sweats, fevers, or weight changes, a malignancy was lower on the differential.

**Figure 1:**
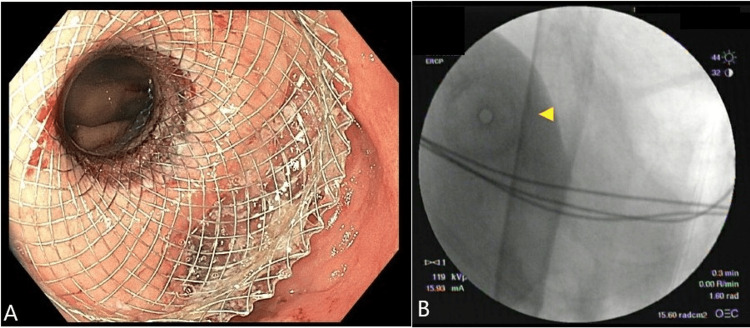
AXIOS Stent Shown in an Endoscopic Ultrasound Shown is an endoscopic ultrasound (EUS) demonstrating an AXIOS stent. Figure 1A shows the AXIOS stent traversing the Roux-en-Y. The arrow in Figure 1B indicates the location of the AXIOS stent, which was deployed between the gastric pouch and excluded stomach

She was subsequently transferred to our hospital for an endoscopic ultrasound-directed transgastric ERCP (EDGE) procedure to localize the bleeding. Endoscopically, there was evidence of a Roux-en-Y anastomosis in the gastric body, surrounded by healthy-appearing mucosa. Diffuse mild inflammation characterized by edema and erythema was found in the gastric body. Two biopsies were obtained with cold forceps for histology on the greater curvature of the gastric antrum, as well as two biopsies at the incisura and two biopsies in the stomach. Pathology for these lesions resulted in mild chronic gastritis, with negative *Helicobacter pylori *infection. The decision was made to create a gastrogastrostomy using the AXIOS stent system (Boston Scientific, Marlborough, MA) between the gastric pouch and the excluded stomach (Figure 1).

**Figure 2:**
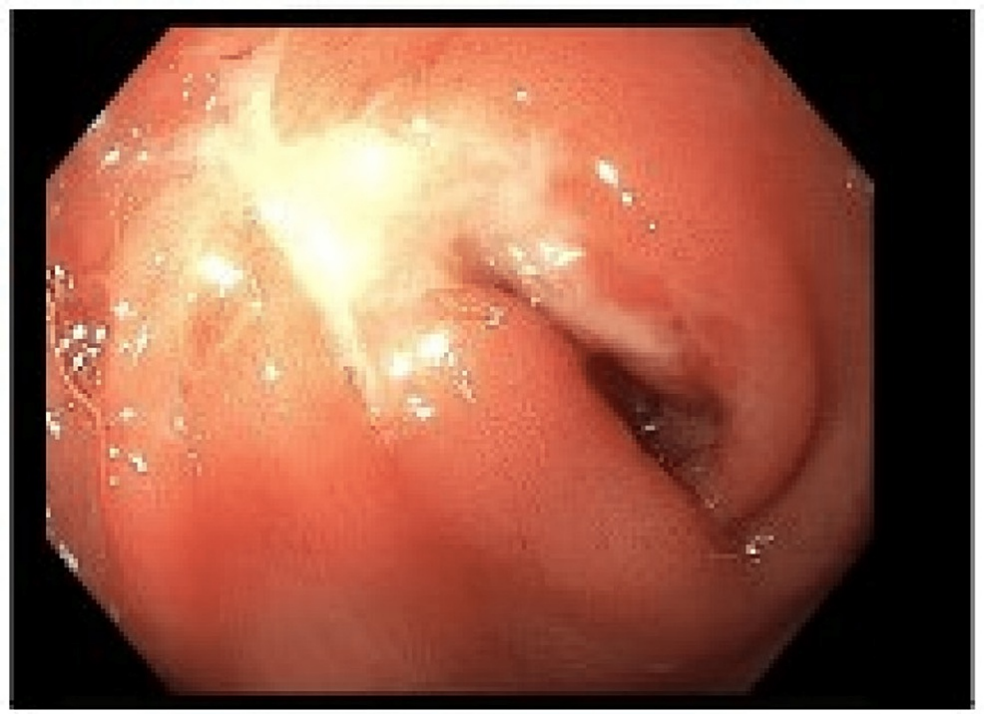
An Ulcerative Mass Diagnosed via Endoscopic Ultrasound-Directed Transgastric ERCP (EDGE) Procedure Shown above is an obstructing ulcerative mass of the gastric antrum in the excluded stomach, visualized during the EDGE procedure ERCP: endoscopic retrograde cholangiopancreatography

Additionally, there were ulcerated gastric deformities in the luminal stenosis found in the gastric antrum. The esophagus and duodenum were macroscopically normal. EDGE demonstrated a hypoechoic ulcerated mass with ill-defined borders in the antrum of the excluded stomach (Figure 2).

**Figure 3:**
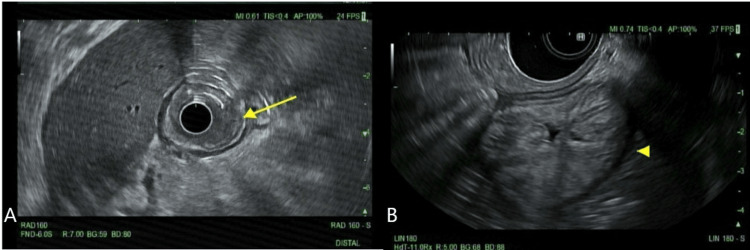
An Ulcerated Mass of the Excluded Gastric Antrum Visualized on Endoscopic Ultrasound (EUS) Shown above are images captured during EUS. The arrows in Figure 3A-3B point to the gastric mass and show endosonographic evidence suggesting invasion into the muscularis propria manifested by abutment

Biopsies of the ulcerative mass was pathologically positive for poorly differentiated adenocarcinoma with signet cell rings. Postoperatively, she was transferred back to the primary hospital to optimize care prior to discharge. Her hemoglobin improved to 9.7 g/dL, and she was discharged with magnesium hydroxide and omeprazole. She returned to our facility for a scheduled EUS with EDGE procedure for staging, approximately one week later. During the second EUS with EDGE, there was visualization of the Roux-en-Y and preexisting AXIOS stent placement with healthy-appearing mucosa and an obstructing (about 80%) gastric antral mass. There was sonographic evidence of invasion in the muscularis propria manifested by abutment (Figure 3).

The pancreas, pancreatic duct, and common bile duct were unremarkable, and there was no pathologic lymphadenopathy. The lesion was endosonographically staged as T2N0. There were no postoperative complications. Upon virtual follow-up two weeks later, she denied pain, melena, fevers, and vomiting and was tolerating an oral diet. She then followed outpatient with oncology for further management.

## Discussion

The incidence of gastric carcinoma varies per geographic region, and although it is on the decline in the United States, it remains to be the third leading cause of death as per the GLOBOCAN data [2,5]. Based on the GLOBOCAN data, the risk of developing gastric cancer up until the age of 74 in males is 1.87% compared to 0.79% in females worldwide [2]. More specifically, one study reported the incidence of gastrointestinal tumors after Roux-en-Y surgery to be 0.8% compared to the general population of 0.0006%-0.0015% [4]. There are various components involved in the progression of gastric carcinoma, including *Helicobacter pylori* and dietary and social habits [1]. The incidence of gastric carcinoma is also rising due to advancements in technology [1].

Despite dieting fads and a stronger push toward “healthy living,” obesity is still problematic particularly in the Western world. Gastric bypass is indicated for those with a body mass index greater than 40 after lifestyle modifications or for those with resistant diabetes [3]. Roux-en-Y is a type of surgical procedure with various anatomical methods to achieve a significant reduction in one’s body fat. This ultimately reduces the morbidities and mortalities associated with obesity [3]. Following Roux-en-Y, it can be difficult to diagnose pathology there with traditional endoscopic modalities [5,7]. Often, a modified endoscopic procedure, known as endoscopic ultrasound-directed transgastric ERCP (EDGE), is utilized for better visualization and diagnostic yield [5-7]. During EDGE, a stent is placed between the excluded stomach and the truncated gastric tract, allowing for adequate visualization and easier access to the distal structures [5,7]. Balloon-assisted duodenoscopes were developed first but were limited in their ability to access the ampulla [6]. Initially, these scopes were only indicated for the use of hepatobiliary disorders following Roux-en-Y surgeries, but in more recent years, other approaches were created to also evaluate the remnant stomach [6]. Laparoscopy-assisted ERCP was developed specifically for this; however, EDGE is a less invasive approach associated with less intraoperative risk and smaller recovery time [7]. Additionally, compared to double-balloon-assisted enteroscopies and laparoscopic-assisted enteroscopies, the EDGE procedure is more cost-effective due to the use of less procedural equipment and has also been shown to result in higher total quality-adjusted life years [6]. If the incidence of gastric cancer in the excluded stomach continues to rise, perhaps a screening tool utilizing EDGE may aid in the earlier diagnosis of malignancy with a less extensive workup [5].

There is a higher incidence of gastric cancer after Roux-en-Y compared to the general population, but rarely, a few cases describe occurrence within the excluded stomach [4,5,7]. One case series described 18 patients with the diagnosis of gastric adenocarcinoma of the excluded stomach after gastric bypass surgery [7]. A Brazilian study evaluated 3,047 patients after Roux-en-Y with gastric cancer; however, only one of which occurred in the excluded stomach [8]. It is thought that there may be physiological changes in the excluded stomach after Roux-en-Y surgeries, making it susceptible to malignant transformation [8]. One specific hypothesis suggests that the presence of pancreaticobiliary reflux leads to prolonged gastritis subsequently increasing the risk of gastric adenocarcinoma [4]. Alternatively, other hypotheses emphasize a potential infectious etiology postoperatively that may go unnoticed, causing a prolonged inflammatory state [4]. It is likely that the etiology is multifactorial.

Like many malignancies, gastric cancer may present with nonspecific signs of weight loss, dysphagia, abdominal pain, and iron deficiency anemia [7]. Our patient presented with melanotic stools and iron deficiency anemia and had an extensive workup with multiple endoscopies and video capsule endoscopy; however, she did not have a clear source of bleeding. It was not until the patient underwent the EDGE procedure where the source was clearly localized and biopsy detected gastric carcinoma. The five-year survival rate for gastric cancer is reported to be 31% in the United States, but given the diagnostic delay and location of the gastric adenocarcinoma, this may not be reflective in our patient [4]. The overall prognosis of gastric malignancy within the excluded stomach is ultimately unknown due to the limited cases documented in existing literature [4].

## Conclusions

Compared to traditional endoscopic approaches and even newer procedures using balloon-assisted, spiral, or laparoscopic-assisted enteroscopy, EDGE is a superior modality to diagnose and treat conditions of the gastrointestinal tract following Roux-en-Y surgeries. Our patient represents a rare entity of gastric adenocarcinoma of the excluded stomach, 20 years after a Roux-en-Y procedure, diagnosed via EDGE after a five-year workup for melena and iron deficiency anemia was inconclusive. It is likely that the etiology of gastric adenocarcinoma after gastric bypass is multifactorial; however, the occurrence of gastric adenocarcinoma in the excluded stomach specifically, following Roux-en-Y, is not well documented. There are only a few case reports currently published on gastric malignancies of the excluded pouch, and the overall prognosis of such malignancies is unknown.
